# L'obésité en consultation cardiologique à Lomé: prévalence et facteurs de risque cardio-vasculaire associés - étude chez 1200 patients

**Published:** 2012-08-08

**Authors:** Soulemane Pessinaba, Komlavi Yayehd, Machiude Pio, Réné Baragou, Yaovi Afassinou, Tchaa Tchérou, Findibé Damorou

**Affiliations:** 1 Faculté Mixte de Médecine et de Pharmacie, Université de Lomé, Togo

**Keywords:** Obésité, prévalence, facteurs de risque, Obesity, prevalence, risk factor

## Abstract

**Introduction:**

Introduction: Les objectifs de ce travail étaient de déterminer la fréquence de l'obésité et celle des autres facteurs de risque cardio-vasculaire chez ces patients obèses à Lomé (Togo).

**Méthodes:**

Il s'est agi d'une étude multicentrique transversale de prévalence. Elle s'est déroulée du 05 septembre 2005 au 04 mars 2006 et a porté sur les malades vus en consultation externe dans 3 services de cardiologie de la commune de Lomé. Ont été inclus dans cette étude les malades ayant un surpoids selon les normes de l'OMS.

**Résultats:**

Parmi 1200 patients vus en consultations, 779 (64,92%) avaient une surcharge pondérale. L’âge moyen était de 49,53 ± 17,24 ans. L'obésité était plus fréquente chez les femmes (79,49%) que chez les hommes (20,51%). Un antécédent d'obésité familiale (61,8%) était le principal facteur favorisant. Les autres facteurs de risque cardio-vasculaire retrouvés étaient: sédentarité (82% vs50% chez les non obèses), hypertension artérielle (54,8% vs 39,2%), alcool (50,9% vs 43,9%), dyslipidémie (34,5% vs 20%), diabète (30,9% vs 10,7%) et tabac (14,1% vs 20,3%). La différence était statistiquement significative entre les deux groupes. Les principales complications cardiovasculaires observées chez les obèses étaient: l'ischémie myocardique (26,7%), l'hypertrophie ventriculaire gauche (46,4%), la dilatation cavitaire cardiaque (30,1%) et les accidents vasculaires cérébraux (7,1%).

**Conclusion:**

L'obésité est un problème de santé publique au Togo. Sa prévalence est très élevée et elle est le plus souvent associée aux autres facteurs de risque cardio-vasculaire. Des mesures préventives doivent être mises en jeu pour lutter contre ce facteur de risque. Mots clés: Obésité, prévalence, facteurs de risque.

## Introduction

L'obésité, autre fois considérée comme l'apanage des pays industrialisés, est aujourd'hui devenue une épidémie mondiale. L'Organisation Mondiale de la Santé (OMS) estime que plus d'un milliard d'adultes dans le monde ont une surcharge pondérale et 300 millions d'entre eux sont obèses [1]. La fréquence de l'obésité ne cesse de croître. Dans les pays industrialisés, la fréquence de l'obésité a connu une augmentation allant de 5% à 10% au cours des dix dernières années [2, 3]. Une progression de la fréquence de l'obésité est également confirmée par quelques travaux réalisés dans les pays africains [4–7]. Au Togo, nous ne disposons pas de données sur l'obésité en milieu cardiologique. Les objectifs de ce travail étaient donc de déterminer la fréquence de l'obésité et celle des autres facteurs de risque cardio-vasculaire chez les obèses en cardiologie à Lomé.

## Méthodes

Il s'agit d'une étude transversale de prévalence allant du 05 septembre 2004 au 04 mars 2006, réalisée dans le service de cardiologie du CHU Campus, l'unité de cardiologie de l'hôpital secondaire de Bè et le cabinet médical Bonne Esperance. Elle a porté sur les patients vus en consultation externe. Etaient inclus tous les patients âgés de 18 ans et plus, ayant un surpoids ou une obésité. Etaient exclus les patients hospitalisés et les patients ayant une cardiopathie congénitale.

Les paramètres étudiés étaient: les données sociodémographiques (âge, sexe, profession), les données cliniques (pression artérielle, poids, taille, tour de taille, tour de hanche, indice de masse corporelle (IMC)) et les autres facteurs de risque cardio-vasculaire (diabète, dyslipidémie, tabac). Le poids a été pris à l'aide d'une pèse personne chez un patient dévêtu. La taille a été mesurée à l'aide d'une toise chez un patient déchaussé. L'IMC était obtenu par le rapport: Poids (Kg)/(taille (m)^2^). La surcharge pondérale était définie par un IMC entre 25 et 29,9 kg/m^2^, l'obésité un IMC entre 30 et 34,9 kg/ m^2^ et l'obésité morbide, un IMC au delà de 35 kg/ m^2^.

Le tour de taille (TT) et le tour de hanche (TH) étaient mesurés à l'aide d'un mètre ruban sur un sujet débout et dévêtu. Lorsque le rapport TT/TH était inférieur à 0,9 chez la femme et 1 chez l'homme, l'obésité était considérée gynoïde et androïde dans le cas inverse [8]. La pression artérielle était mesurée à l'aide d'un sphygmomanomètre, sur un patient allongé après au moins dix minutes de repos. Trois contrôles ont été effectués lorsque les premiers chiffres tensionnels étaient élevés. La sédentarité était définie par l'absence d'activité physique quotidienne ou une activité physique d'une durée < 150 mn par semaine. Pour la biologie, les normes retenues étaient une glycémie < 1,26g/l, un taux de cholestérol total < 2g/l, un taux de LDL-cholestérol < 1,6 g/l, un taux de triglycérides < 1,5g/l. La glycémie était contrôlée chez les individus présentant un premier dosage > 1,26g/l.

Les données recueillies ont été saisies et analysées avec le logiciel Epi info version 3.5.1. Les tests de Khi 2 (Pearson et Yates) pour les comparaisons de proportions et de Student pour les comparaisons de moyenne et la régression logistique ont été utilisés. Le seuil de significativité était retenu pour une valeur de p < 0,05.

## Résultats

Pendant la période d’étude, 1200 patients avaient été enregistrés dont 779 (64,9%) avaient un excès pondéral et 395 (32,9%) avaient une obésité. Il s'agissait de 325 hommes (41,7%) et 454 femmes (58,3%). Le sexe ratio était de 0,72. Trois cent quatre vingt quatre (49,3%) avaient un surpoids, 332 (42,6%), une obésité et 63 (5,3%), une obésité morbide. Parmi les obèses (395), on notait 314 femmes (79,5%) et 81 hommes (20,5%). L’âge moyen des patients était de 49,53 ± 17,24 ans (extrêmes 18 et 97ans). Deux cent treize patients (27,3%) avaient moins de 40 ans, 347 (44,5%) avaient entre 40 et 59 ans et 219 (28,1%) avaient plus de 59 ans. Sur les 395 patients obèses, 16 (4,1%) avaient entre 18 et 39 ans, 212 (53,7%) avaient entre 40 et 59 ans et 167 (42,3%) avaient 60 ans ou plus.

Dans le Tableau 1, nous avons représenté les caractéristiques de la population générale. La plupart de nos patients (85,8%) habitaient Lomé. Seuls 111 patients habitaient d'autres villes du pays. Parmi les 779 patients, 365 (46,9%) n’étaient pas scolarisés, 235 patients (30,2%) avaient un niveau scolaire inférieur au baccalauréat, 60 patients (7,7%) avaient le baccalauréat et 119 patients (15,3%) avaient effectué des études universitaires.

**Tableau 1:** Caractéristiques générales de la population d’étude (n = 1200)

	Total	Femmes	Hommes
Effectif	1200	624 (52%)	576 (48%)
Age moyen (ans)	48±4	48±3	48±6
Poids moyen (kg)	72,6±15	70,7±10	75,1±17
Taille moyenne (cm)	164±9	159±7	170±8
IMC moyen (kg/m^2^)	27±4,2	28±4,2	26±4,2
PAS moyenne (mmHg)	135±15,20	137±16,18	136±15,70
PAD moyenne (mmHg)	85±9,22	83±10,34	85±9,60

PAS : pression artérielle systolique; PAD : pression artérielle diastolique; IMC : indice de masse corporel

**Figure 1:**
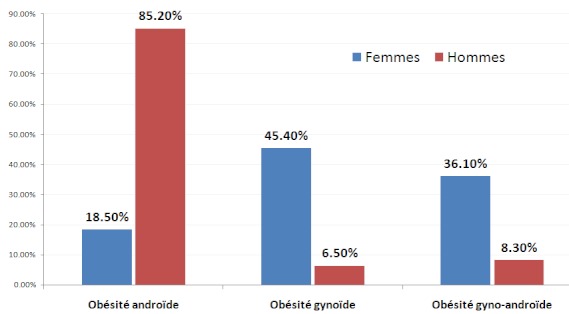
Répartition des sujets en fonction du sexe et du type d'obésité

Concernant le type d'obésité, 227 (29,1%) avaient une obésité gynoïde, 361 (46,3%) une obésité androïde et 191 (24,5%) une obésité gyno-androïde. La majorité des hommes (227 soit 85,2%) avaient une obésité androïde et la majorité des femmes (206 soit 45,4%) avaient une obésité gynoïde (Figure 1).

Les antécédents familiaux retrouvés étaient: obésité (61,8%), HTA (48,5%), diabète (15,4%), HTA et diabète (8,1%), hémoglobinopathie (6,4%) et goutte (1,5%). L'obésité était associée à des proportions variables aux autres facteurs de risque cardio-vasculaire. Il s'agissait de la sédentarité (82%) contre 50% chez les non obèses (p <0,0001), de l'hypertension artérielle (54,8% vs 39,2% p = 0,0000002), de la consommation de l'alcool (50,9% vs 43,9% p = 0,04), de la dyslipidémie (34,5% vs 20% p = 0,0000001), du diabète (30,9% 10,7% p < 0,0001) et du tabac (14,1% vs 20,3% p = 0,006). La différence était statistiquement significative entre les deux groupes pour tous les facteurs de risque associés. La prévalence du syndrome métabolique était de 14,9%. Le Tableau 2 représente certaines caractéristiques des sujets obèses et non obèses.

**Tableau 2:** Valeurs moyennes de la pression artérielle, de l'Indice de Masse Corporelle (IMC) et du bilan biologique des sujets en surcharge pondérale et sans surcharge pondérale (n = 1200)

	Surcharge pondérale	Poids normal	p
PAS moyenne (mmHg)	148,4±23,3	130±20,5	<0,001
PAD moyenne (mmHg)	85,4±13,3	82,4±12,5	<0,001
IMC moyen (kg/m^2^)	30±4,8	23±1,8	<0,001
Cholestérol total (g/l)	2,70	1,80	0,001
LDL cholestérol (g/l)	1,90	1,40	0,002
HDL cholestérol (g/l)	0,46	0,58	0,0001
Triglycéride (g/l)	1,43	1,17	0,43
Glycémie moyenne (g/l)	1,35	1,1	0,01

PAS : pression artérielle systolique; PAD : pression artérielle diastolique; IMC : index de masse corporel

Les complications cardiaques retrouvées chez les obèses étaient: l'ischémie myocardique (26,7%), l'hypertrophie du ventricule gauche (46,4%), la dilatation de cavités cardiaques (30,1%) et les accidents vasculaires cérébraux (7,1%).

## Discussion

L’étude de l'obésité et des autres facteurs de risque cardio-vasculaire est d'un grand intérêt dans nos pays en développement. En effet des études avaient montré la progression constante de l'obésité depuis les années 90, aussi bien dans les pays développés [9, 10] que dans les pays en voie de développement [11]. La prévalence de la surcharge pondérale dans notre étude était proche de celle des autres études africaines [4–6]. Une prédominance féminine de l'obésité a été soulignée dans notre étude (79,5% contre 20,5%). Même si certains auteurs [12, 13] avaient noté une prédominance masculine, la plupart des études retrouvent une prédominance des femmes dans l'obésité [4, 14, 15]. Près de la moitié (46,9%) des sujets ayant un excès de poids dans notre étude n’était pas scolarisée. L'obésité était également plus fréquente chez les patients ayant un niveau d'instruction bas (non scolarisé et primaire), selon les travaux de Monteiro au Brésil [16].

L'obésité est une maladie chronique d’étiologies multiples, incluant la génétique, l'environnement, le mode de vie et l'alimentation. De nombreuses études épidémiologiques ont montré le rôle de l'obésité comme facteur de risque indépendant de maladies cardio-vasculaires [17]. Elle constitue également un facteur de risque d'autres maladies qui sont-elles mêmes facteurs de risque de maladies cardio-vasculaires à savoir: le diabète, la dyslipidémie et l'hypertension artérielle [18]. Dans note étude la prévalence du diabète, de l'hypertension artérielle et de l'hyper LDL cholestérol chez les obèses était plus élevée que celle chez les sujets non obèses avec une différence statistiquement significative. L'hypertension artérielle et le diabète de type 2 étaient également fortement associés à l'obésité abdominale dans le travail de Pouchain [19]. La présence de ces facteurs de risque chez le sujet obèse doit faire rechercher un syndrome métabolique, facteur de risque majeur de maladies cardio-vasculaires [20]. Nous avons noté 14,9% de syndrome métabolique chez nos sujets obèses. Le bénéfice d'une activité physique régulière sur la santé en générale a été bien démontré [21]. La sédentarité multiplie par deux le risque de développer une maladie cardio-vasculaire [22]. La sédentarité était significativement associée à l'obésité dans notre étude. L'effet protecteur de l'activité physique sur les maladies cardio-vasculaires s'explique par le fait qu'elle entraine une diminution du poids corporel, une baisse de la pression artérielle, une augmentation du taux de HDL, une baisse des triglycérides, une augmentation de la tolérance au glucose et une baisse du niveau de la glycémie [23]. L'obésité était significativement associée aux autres facteurs de risque cardio-vasculaire sauf pour le tabagisme. Ce qui faisait de nos sujets obèses, des patients à haut risque cardio-vasculaire comme en témoin les complications cardio-vasculaires retrouvées chez ceux-ci.

## Conclusion

L'obésité est un problème de santé publique au Togo. Sa prévalence est très élevée en consultation cardiologique. Elle est le plus souvent associée à d'autres facteurs de risque cardio-vasculaire, faisant des patients obèses des sujets à haut risque. La prévention serait le moyen le plus efficace pour lutter contre l'obésité. Cette prévention doit se faire sur l'adoption de saines habitudes alimentaires et de la pratique d'une activité physique régulière.
